# Preparation of a polyaniline/ZnO-NPs composite for the visible-light-driven hydrogen generation

**DOI:** 10.1038/s41598-024-53672-2

**Published:** 2024-02-07

**Authors:** Rasoul Azmayesh, Hamid Naghshara, Sajedeh Mohammadi Aref, Mohammad Ghafouri

**Affiliations:** 1 Faculty of Physics, University of Tabriz, Tabriz, Iran; 2 Research Institute of Applied Physics and Astronomy, University of Tabriz, Tabriz, Iran; 3 Physics Department, Islamic Azad University, Shabestar Branch, Shabestar, Iran

**Keywords:** Physics, Applied physics, Condensed-matter physics

## Abstract

Compositions of ZnO nanoparticles and polyaniline, in the form of emeraldine salt, were manufactured as thin layers by using the spin-coating method. Then, the effect of polyaniline content on their photoelectrochemical characteristics was studied. Results indicate that all the samples are sensitive to light. Besides, with 0.30% of PANI, the composite sample demonstrates the highest photocurrent density; also, its photocurrent increment starts to increase at a voltage of ⁓ 1.23 V (vs. RHE), which is approximately in accordance with the theoretical potential of water electrolysis. Furthermore, since the rate of electron–hole recombination in this composite sample is the lowest, it possesses the highest photoelectrochemical efficiency. Main findings were analyzed with respect to UV–visible absorption and photoluminescence spectra as well as SEM micrographs of the samples and Raman spectral measurements. Besides, electrochemical impedance spectroscopy analysis was applied to both pure ZnO and the sample with the best response. Effects of drying temperature and layer thickness were also investigated.

## Introduction

At the present time, the unbridled growth of industrialization and population, the growing requirement of available energies and their increasing costs have made the use of solar energy highly necessary^1^. The solar energy that irradiates the surface of the Earth (1.3 × 10^5^ TW) exceeds the current global human energy consumption (1.6 × 10^1^ TW in 2010) by roughly four orders of magnitude^2^. Also, as eco-friendly energy, solar energy is accessible and consistent and can be exploited thermally, electrically, and chemically^3–5^. To researchers who pursue the aim of obtaining optimal use of solar energy, two approaches are of great interest: solar cells and hydrogen production through water splitting, both of which employ a roughly similar mechanism. This mechanism can be summarized as the absorption of a photon in an appropriate semiconductor, electron-holes production (and their subsequent separation via special methods), and the final hole consumption in order to produce electrical current or water splitting. Interestingly, water splitting via solar energy irradiation has drawn much attention to the production of renewable hydrogen as a source of clean energy from water on a large scale^6,7^. In other words, the evolution of photoelectrocatalytic hydrogen through solar energy consumption has received tremendous attention since it is the most promising way towards large-scale production of hydrogen in the future^6,7^. Solar hydrogen will be significant in prospective sustainable energy societies since it is storable and, more importantly, transportable. Moreover, it can be efficiently converted into electricity by using fuel cells whenever necessary. Furthermore, hydrogen is applicable as feedstock in the modern chemical industry. It could be used for recycling carbon dioxide via chemical processes such as the Fischer–Tropsch reaction and methanol synthesis^8^.

Water splitting reaction is an uphill reaction in which the Gibbs free energy increases by 237 kJ/mol. The energy needed to drive photo-catalytic and photoelectrochemical (PEC) water splitting is provided by either light or, ideally, sunlight. To increase the efficiency of a water splitting system, it is necessary to inhibit the electron–hole recombination process. One way to force electrons and holes to transfer is “adjusting the Fermi energy level” through proper band alignment between junction semiconductors, which can inhibit the excited carriers’ accumulation inside semiconductors; this process, in turn, reduces recombination^9^. Photo-excited charge carriers, produced in the principal semiconductor, are extracted and, subsequently, move to the added material or substrate; hence, their accumulation within the principal semiconductor does not occur. As a result, e–h recombination is inhibited, and the cell’s efficiency enhances. Some reports of the added materials, which inhibit recombination by different mechanisms, are Ni_2_P, polyaniline (PANI), carbon, etc.^10,11^. A more recent approach to prevent e–h recombination is manipulating nanostructures, i.e., employing grain boundary’s special characteristics with high mobility of electrons and holes to increase the cell’s efficiency^12^. This means that the rapid transfer of electrons and holes between grains and grain boundaries or into the substrate, as well as their consumption in a redox process, prevents e–h recombination due to a lack of electrons’ and holes’ accumulation in photo-catalytic semiconductors. According to the literature, despite various reports on many types of photo-catalytic semiconductor materials^13–15^, there have been few detailed studies on photo-catalytic properties accompanied by nanostructural transitions during the process. Besides, due to their chemical stability, wide band gap semiconductors such as BaTiO_3_, SnO_2,_ and Ag_3_PO_4_ are used in photoelectrochemical conversion^16–18^. However, their large gap (E_g_), exceeding 3 eV, makes them unabsorptive for solar irradiation. Nowadays, much research is devoted to organic semiconductors which have further advantages such as environmental safety, easy synthesis, and light weight. Conductive polymers, among which polyaniline is a promising case, are also considered to be effective materials for use in photoelectrochemical devices. Polyaniline is a low-cost polymer with an optical gap of ∼ 1.8 eV that allows the conversion of a large part of sunlight. It has displayed its efficiency in photodegradation and hydrogen production^19,20^.

Nowadays, there are various promising and creative ways to produce hydrogen using water splitting methods, e.g., employing different types of metal oxides^21^ as well as GaN nanowire-based photoanodes^22^. In this regard, studies have shown that ZnO exhibits chemical stability and large exciton binding energy; it can also be synthesized via different simple and inexpensive methods^23,24^. In order to improve the properties of photoelectrochemical devices, a new approach based on ZnO-PANI composition has been reported^25^. According to the literature, the PANI-ZnO hetero-system has been successfully used in the degradation of organic pollutants, in solution-treated photo-detectors, and in the photovoltaic performance of polymer solar cells^26–28^. In addition to these methods, there are coupled processes that are interesting from a practical point of view. For example, there are different reported uses of polyaniline-ZnO nanoparticles as treating industrial effluents and organic pollutants of the air^29^.

This research intends to introduce a water splitting system with optimum efficiency achieved through using various amounts of PANI in “ZnO-NPs/PANI” composites. To do so, ZnO nanoparticles and de-doped PANI were synthesized via the Beek method and McDiarmid procedure, respectively. Then, ZnO nanoparticles and PANI were separately dissolved in chloroform and NMP at specific concentrations of 200 mg/mL and 5 mg/mL, respectively. Five composite samples were prepared at different concentrations of ZnO-NPs and PANI via the spin-coating process. Eventually, the effect of PANI percentage on systems’ efficiency was examined with regard to time dependency of photocurrent density, photocurrent density as a function of voltage versus the reversible hydrogen electrode (RHE), and UV–visible spectrum of all composite samples. Results indicate that the 0.30%-PANI sample has the best efficiency. Afterwards, the photoluminescence and electrochemical impedance spectra of the best sample were compared with those of pure ZnO. Then, the effect of drying temperature at temperatures of 50 °C, 100 °C, and 150 °C, and the effect of layers’ thickness on the 0.30%-PANI sample were studied. Finally, Raman spectral measurements were used to gain insights into any structural changes.

According to literature, there are a variety of reports on the benefits of ZnO/PANI composite in enhancing the water splitting efficiency^30–32^. As a first attempt to use a photo-anode to generate hydrogen, this study is undoubtedly original and yields important results. Indeed, its simple and low-cost method, during which PANI does not penetrate the ZnO-NPs structure, is obviously unique. Practically, in contrast with some previous studies^33^, here ZnO-NPs serves as the base of the cell whose efficiency is improved by adding PANI. Actually, the composite has a grain/grain-boundary structure. Also, this study introduces an optimum amount of PANI (0.30%) whose effect on the cell’s efficiency is the highest. In fact, polyaniline facilitates the exciton production via effective light absorption; it also displays impressive charge separation, compared with pure ZnO-NPs.

## Material and methods

### Synthesis of zinc oxide nanoparticles

In line with the method proposed by Beek et al.^34^, first 1.5 g (26 mmol) of potassium hydroxide (Merck, ≥ 85.0%) was dissolved in 65 mL of methanol (Merck, ≥ 99.9%) at room temperature; a magnetic mixer was used to perform the process of stirring for 15 min,. While the stirrer rotated strenuously, 3 g (14 mmol) of zinc acetate dihydrate (Merck-108802, ≥ 99.5%) was dissolved in 125 mL of methanol at a temperature of 60 °C for 5 min. Afterwards, inside a closed system that prevents the evaporation of volatile alcohol, the first solution was added dropwise to the second one—also subjected to strenuous rotation—in 10 min at a temperature of 60 °C. Then, the final solution was stirred for 3 h at a temperature of 60 °C. Afterwards, the synthesized particles were slowly cooled and settled down simultaneously. After the complete sedimentation of the particles, the liquid above the settled particles was drained; the process was followed by washing and centrifuging the nanoparticles with methanol twice. Finally, the produced nanoparticles were gradually dissolved in chloroform (Merck, ≥ 99.8%) with a concentration of 200 mg/mL, which resulted in a translucent solution.

### Synthesis of polyaniline

According to the McDiarmid method, de-doped polyaniline in the form of emeraldine salt was synthesized from aniline monomer^35^. The synthesized polyaniline, in the form of a dark navy blue powder, was gradually dissolved in NMP (Merck, ≥ 99.0%) to hand in a 5 mg/mL solution. The final solution was filtered using a PTFE-0.45 μm filter to obtain particles with a diameter lower than 0.45 μm.

### Preparation of “ZnO-NPs/PANI” composites

At this stage, five different samples at various concentrations of ZnO-NPs and PANI were prepared according to Table 1. To do so, the selected amounts of “ZnO-NPs dissolved in chloroform” and “PANI dissolved in NMP” solutions were stirred together for 5 s, and then the final product was spin-coated on ITO substrate at 1500 rpm/min followed by drying at a temperature of 100 °C, to hand in 5 different layers with a thickness of ⁓ 700 nm.

**Table 1:** The amounts of various concentrations of ZnO-NPs and PANI, and the samples’ name.

No.	Amount of ZnO-NPs solution (mL)	Amount of PANI solution (mL)	ZnO-NPs concentration (%)	PANI concentration (%)	Sample’s name
1	0.2	0	100	0	0.00%-PANI
2	0.2	0.0125	99.85	0.15	0.15%-PANI
3	0.2	0.025	99.70	0.30	0.30%-PANI
4	0.2	0.0375	99.55	0.45	0.45%-PANI
5	0.2	0.05	99.40	0.60	0.60%-PANI

### Investigation of irradiation-dependent properties

In order to study the photoelectrochemical (PEC) properties of the cells, 1 M KOH aqueous solution was used as an electrolyte; it was necessary to avoid the effects of any impurities and provide impurity-independent results. Besides, electrochemical measurements were carried out in an autolab PGSTAT100, Eco Chemie potentiostat/galvanostat. A halogen lamp (100 W) was used as a visible-light illuminating source; the front illumination was used and the system had been calibrated regarding simulated AM 1.5G illumination. A formal three-electrode photoelectrochemical cell including the composites as working electrodes with an active area of 1 cm^2^, platinum wire as the counter electrode, and saturated calomel electrode (SCE) as the reference electrode were used in experiments. Thereupon, the different stages were carried out as follows:

1. To emphasize that ZnO nanoparticles are successfully synthesized, the structural properties of the 0.00%-PANI sample were investigated by XRD (Siemens D500, Germany, by CuKα radiation, λ = 1.540Å) and SEM (Tescan, MIRA3, FEGSEM, Czech).
2. “Time dependency of photocurrent density” and “a proof of sensitivity of the samples to light applying on/off diagram of the two-electrode test” were verified.
3. Photocurrent density as a function of voltage versus the reversible hydrogen electrode (RHE) and the related dark-photo currents, as well as the on/off diagram, were studied.
4. The UV–visible absorption spectrum of the samples was recorded for the synthesized and composite samples using a PHARMA spectrophotometer, SHIMADZU model 1700.
5. The photoluminescence spectrum of pure ZnO and the best sample were obtained using a PL Spectrophotometer (JASCO, FP-6200, America). Also, their electrochemical impedance spectroscopy analysis was registered via a KC-605 LCR meter, made by Japan. The EIS measurement was carried out covering a frequency interval of 44–10^5^ Hz; the voltage range of the LCR meter was 0–0.5 V, which was applied automatically to the sample. The optimum values of the drying temperature, as well as layers’ thickness, were also investigated for three different samples (all of which had the PANI concentration of 0.30%). SEM micrographs of the well-behaved composite, as well as its EDS mapping, were also obtained using a Cam Scan MV 2300, made by the Czech Republic. Needless to say, all the measurements were performed at room temperature.
6. The Raman spectra were measured at a temperature of − 40 °C in the wavelength range of 300–1600 cm^−1^, using the 566.197 nm excitation lines from an Ar ion laser and a UniDron-A spectrometer, made by Korea.

## Results and discussion

**Figure 1:**
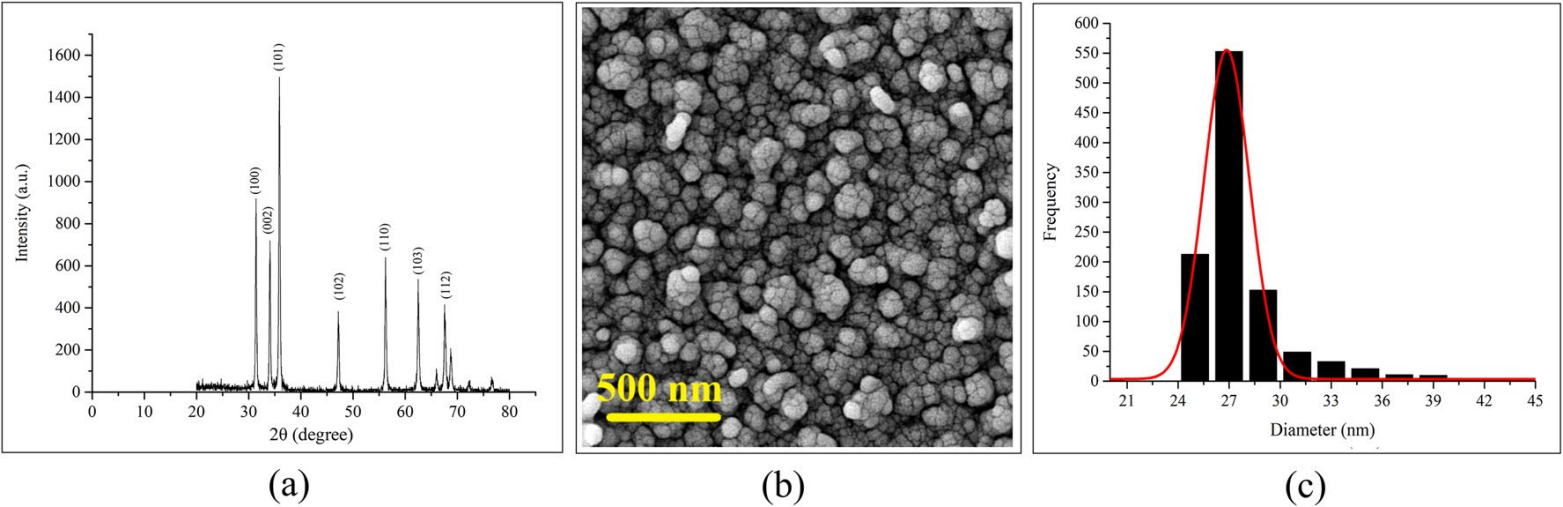
(**a**) XRD pattern, (**b**) SEM micrograph, and (**c**) particle size distribution diagram of 0.00%-PANI sample.

Figure 1 shows the XRD pattern and the SEM micrograph of the 0.00%-PANI sample. The XRD spectrum (Fig. 1a) indicates that its major diffraction peaks can be well indexed to hexagonal-phase ZnO—compared with the standard diffraction pattern (JCPDS Card 00-009-0432)—hence confirming the successful synthesis of pure ZnO nanoparticles. Another application of XRD patterns is to calculate the size of ZnO nanoparticles using the Scherrer model defined as follows^36^:

$$ {\text{D}} = 0.{9 }\lambda /\beta_{{{2}\theta }} {\text{cos }}\left( {\theta_{{\text{B}}} } \right) $$

Based on this relation and the width of the prominent peaks of the XRD pattern, the average crystallite size of the ZnO nanostructured film is calculated as 28.3 nm. With respect to the SEM micrograph (Fig. 1b) and based on the particle size distribution diagram (Fig. 1c), it is possible to recognize that ZnO nanoparticles are spherical and have a mean size of 26.86 ± 2.67 nm. In fact, these particles are conglomerated in grain shapes of nearly uniform sizes.

**Figure 2:**
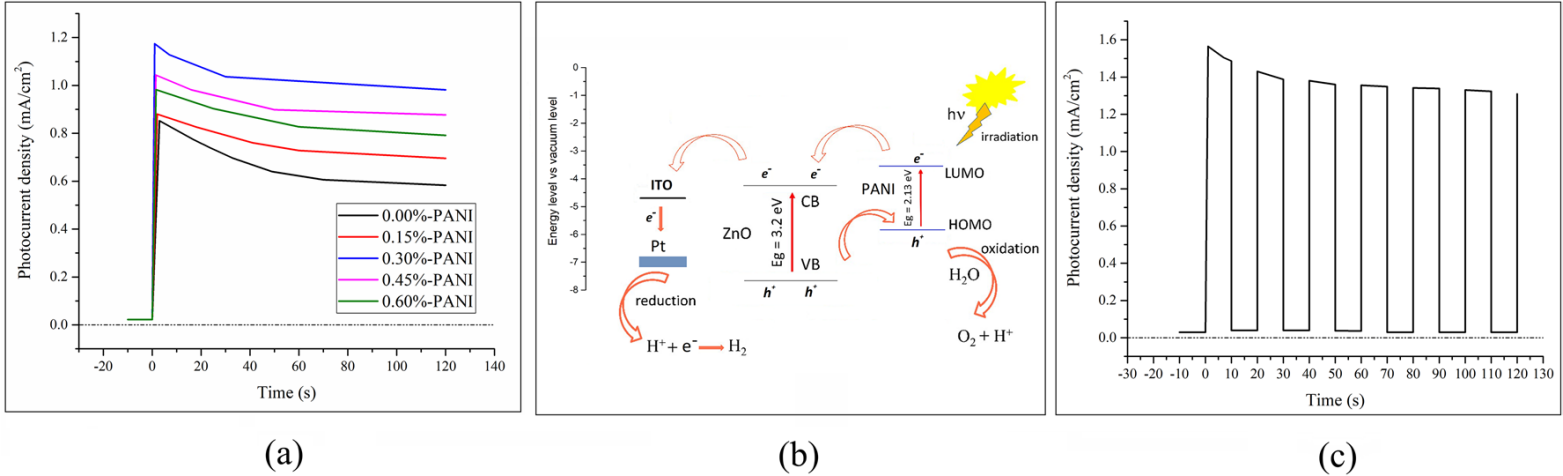
(**a**) The two electrode “photocurrent density- time” diagrams of all the samples, (**b**) Schematic of charge transformation between zinc oxide nanoparticles and PANI in order to interpret water splitting via PEC cells, and (**c**) The on/off diagram of the two-electrode test for the 0.30%-PANI sample, with dark–light duration of 10 s.

To get an accurate estimate of a PEC cell’s efficiency, it is necessary to employ a two-electrode system to measure the photocurrent as a function of time. According to the two-electrode “photocurrent density- time” diagram (Fig. 2a), when samples are subjected to light, photocurrent density rises dramatically to reach its maximum. It is then followed by a gentle decrease to achieve a steady state. The decay is due to the presence of surface traps in the material, which leads to charge trapping^37^. Then, the current density of all the samples remains almost stable, even after several minutes of exposure to light; this is the proof of the good photo-stability of the composites. Also, no significant decrease occurs in their activity, which indicates their high electrochemical stability. However, the efficiency of the electrodes decreases as a function of time in such a way that after 8 days, the cells’ efficiency reaches about 70% of the initial value. Furthermore, the addition of polyaniline generally increases photocurrent density. Interestingly, the 0.30%-PANI sample yields the highest photocurrent. To explain this behavior, it can be said that when a cell is exposed to radiation, excitons are produced, which should be separated to electrons and holes before recombination. The common strategy to separate a Frenkel exciton is the utilization of an inhomogeneous interface whereby an electron could transfer to an acceptor. In these hybrid cells, when an exciton is formed as a result of a photon’s absorption, it can be separated to an electron and a hole at the “ZnO-NPs/PANI” inhomogeneous interface. As the conduction band of zinc oxide nanoparticles is lower than the LUMO level of polyaniline, electrons transfer to ZnO and then—after being collected by ITO substrate—reach the Pt cathode through the external circuit (Fig. 2b). It is noteworthy that the CB position of ZnO is about 4.19 eV with a bandgap of about 3.20 eV (using vacuum level as a reference)^38^, and the calculated work function of PANI is 4.42 eV with a bandgap of about 2.13 eV^15,39^. Besides, holes transfer to PANI—since its HOMO level is higher than the valance band of ZnO – and are directly consumed by water. It should be noted that PANI increases the chance of e–h separation because it forms an inhomogeneous grain boundary between ZnO-NPs. Therefore, the addition of PANI results in higher photocurrent density. On the other hand, since it has been dehydrogenated with ammonia, it seems that a further increase of PANI prevents both electrons’ and holes’ transfer at the inhomogeneous interface. To a great extent, this may indeed be attributed to the agglomeration of PANI particles. Accordingly, in case of higher PANI percentages, the resistance of the intergranular layer, the probability of exciton’s electron, and hole recombination increase. It is noteworthy that PANI does not penetrate the ZnO structure as the sintering temperature is not too high; it just remains at the intergranular area. Also, since the cells’ efficiency is directly proportional to photocurrent density, the 0.30%-PANI sample possesses the highest efficiency. Besides, the on/off diagram of the two-electrode test for this sample, with a dark–light duration of 10 s, indicates the sensitivity of the sample to light (Fig. 2c).

According to literature, coulometry can be used in estimating the amount of produced hydrogen gas. To do so, the calculation of the area under “photocurrent density- time” diagram would be useful. Regarding the electrical current definition, this area is equal to the charge amount as follows^40^:

$$Q={\int }_{0}^{{t}_{r}}i\left(t\right)dt$$

Then, the amount of Q could be used in the following equation which is known as coulometry equation:

$$n= \frac{{W}_{m}}{W}\frac{Q}{F}$$

where n is the number of electrons participating in the electrode process. Also, W_m_ and W are the molar mass and the mass of the electrolyte, respectively. Additionally, F is Faraday's constant which equals:

$$F={N}_{A} \times e\approx 9.65 \times {10}^{4} \, {\text{C}}/{\text{mol}}$$

Here, N_A_ is the Avogadro constant and e is the elementary charge. In this study, W_m_/W is chosen as 0.1 mol^−1^. It is well known that four electrons are equivalent to two hydrogen molecules, and mass and mass density of hydrogen molecule are 3.32 × 10^–24^ g and 0.08988 g/L, respectively. The amounts of produced hydrogen gas are summarized in Table 2.

**Table 2:** The amount of produced H2 gas and the efficiency of the composite samples.

No.	Sample’s name	The amount of H_2(g)_ (µL)	Efficiency vs. 0.00%-PANI
1	0.00%-PANI	0.87	1
2	0.15%-PANI	1	1.15
3	0.30%-PANI	1.39	1.60
4	0.45%-PANI	1.23	1.42
5	0.60%-PANI	1.14	1.31

Moreover, the potential of the working electrode cannot be precisely measured in the simple two-electrode configuration. The purpose of the two-electrode measurement is to assess the efficiency of a PEC cell, taking over-potential loss in both working and counter electrodes into account. When an external voltage is applied to PEC systems, the electrical energy has to be subtracted from the energy gain. For two-electrode measurements, the applied bias photon-to-current efficiency (ABPE) is frequently used. ABPE is defined as:

$$ABPE= \frac{\left|j\right| \times \left({V}_{th}- {V}_{bias}\right)}{{P}_{sun}}$$

Here j, Vth, and V_bias_ are the photocurrent density, the theoretical water-electrolysis voltage (1.23 V), and the applied voltage, respectively. Thus, it is possible to calculate the efficiency of the samples with respect to the efficiency of pure ZnO according to the following equation:

$$\frac{{(ABPE)}_{sample \, with \, PANI}}{{\left(ABPE\right)}_{Pure \, ZnO}}=\left|\frac{{j}_{sample \, \, with \, \, PANI}}{{j}_{ZnO}}\right|$$

Therefore, taking the amount of Q and the test’s time interval into consideration (|j|= Q/t), the photocurrent density can be calculated. Results are summarized in Table 2.

**Figure 3:**
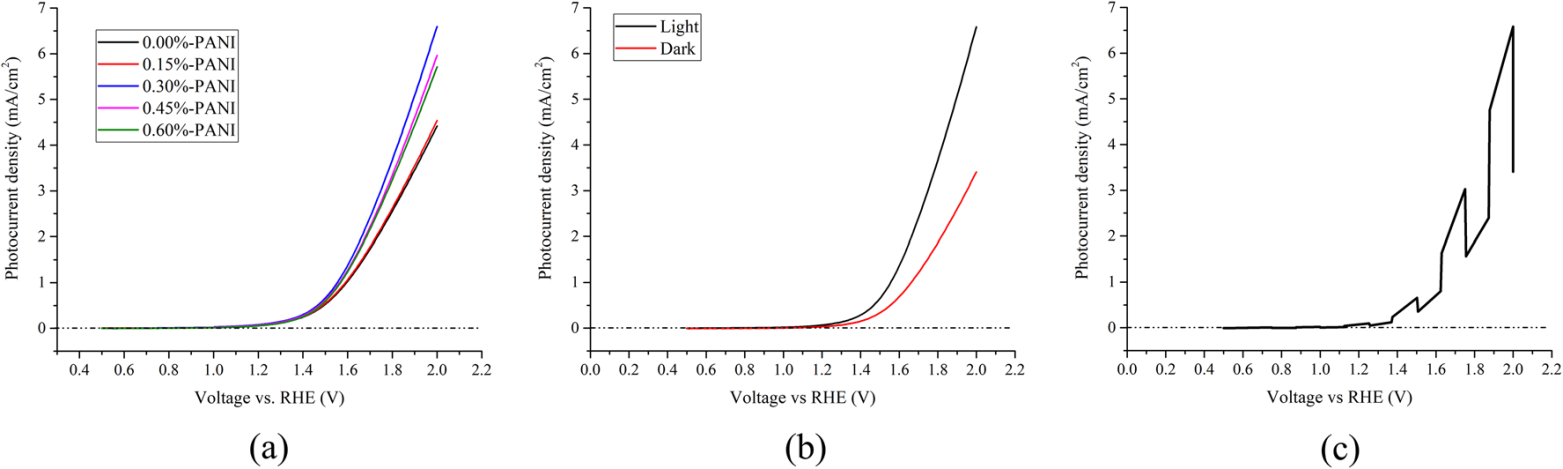
(**a**) Photocurrent density as a function of voltage versus the reversible hydrogen electrode (RHE) for all the samples, (**b**) A comparison of dark and light diagrams for the 0.30%-PANI sample, and (**c**). The on/off diagram of the three-electrode test per 0.125 V for the 0.30%-PANI sample.

The three-electrode test, consisting of composites as the working electrode, platinum as the counter electrode, and saturated calomel electrode (SCE) as the reference electrode, was performed under the radiation of sunlight. As evident in Fig. 3a, photocurrent density is nearly zero for all samples up to ⁓ 1.2 V (vs. RHE). When the voltage approaches ⁓ 1.2 V, however, photocurrent density increases at a relatively significant slope. Again, the 0.30%-PANI sample yields the highest photocurrent, its diagram represents the most significant slope, and the relative photocurrent increment starts to increase at a voltage of ⁓ 1.23 V. This result is crucial since it is well-known that the theoretical potential of water electrolysis is 1.23 V (vs. RHE).

Moreover, a comparison of dark and light diagrams of the 0.30%-PANI sample (Fig. 3b) reveals that under radiation, the starting potential of photocurrent ascent gets closer to 1.23 V. As expected, these results are in accordance with photo anodes’ behavior^41^. According to Fig. 3b, dark-current increases dramatically when voltage reaches ⁓ 1.3 V; In other words, dark current starts at ⁓ 1.3 V. Therefore, at potentials higher than 1.3 V (vs. RHE), the oxygen evolution process is performed effectively. The relatively small current, which is related to lower potentials (vs. RHE), is called the remaining current. Again, the photo-current is significantly high and starts at a potential lower than the oxygen evolution potential (1.23 V vs. RHE); therefore, photo and dark-current diagrams differ from each other. Furthermore, three-electrode measurement was applied at on/off condition per 0.125 V (Fig. 3c). It is noteworthy that approximately similar results were reported for ZnO-based cells using electrolytes such as Na_2_SO_4_ solution^42^. Another obtained result is that applying high potential, i.e., more than 2 V, is bound to lead to material oxidation. Practically, oxidation happens above 2 V because of the high dark current, which seems to get better with light.

As demonstrated, and according to literature, there are variety of materials which have been used as photo-electrodes. In Table 3, a number of previous studies on ZnO and PANI based photo-electrodes are shown. Here, their photocurrent densities, as indicative of their efficiency, are compared. Among these photo-electrodes, our ZnO-NPs/PANI photo-anode can compete with the reported ones.

**Table 3:** Comparison of the photocurrent density of ZnO-NPs/PANI composite with the earlier reported results.

Photo-electrode	Fabrication method	Light irradiation	Type of electrolyte	Photocurrent density	References
PANI/ZnS/ZnO	Hydrothermal	Visible	0.1 M Na_2_SO_4_	0.60 mA/cm^2^ (at 1 V vs. Ag/AgCl)	^30^
ZnIn_2_S_2_/PANI	Hydrothermal	Visible	0.5 M Na_2_SO_4_	0.26 mA/cm^2^ (at 0.8 V vs. Ag/AgCl)	^43^
Al-doped ZnO	Hydrothermal	Visible	1 M KOH	8 μA/cm^2^	^44^
ZnO/ZnS	Hydrothermal	Visible	0.25 M Na_2_S + 0.35 M Na_2_SO_4_	0.12 mA/cm^2^ (at 0.4 V vs. Ag/AgCl)	^45^
PANI/ZnO	Chemisorption	Visible	0.5 M Na_2_SO_4_	0.8 mA/cm^2^ (at 1 V vs. Ag/AgCl)	^31^
Ni-doping/ZnO	Hydrothermal	Visible	0.1 M KOH	1.2 mA/cm^2^ (at 0.5 V vs. Ag/AgCl)	^46^
PANI/ZnO-nanorods	Hydrothermal	Visible	0.1 M Na_2_SO_4_	12 μA/cm^2^ (Zero volt vs. Ag/AgCl)	^32^
ZnO-NPs/PANI	Hydrothermal	Visible	1 M KOH	1.1 mA/cm^2^ (No bias)	Present work

**Figure 4:**
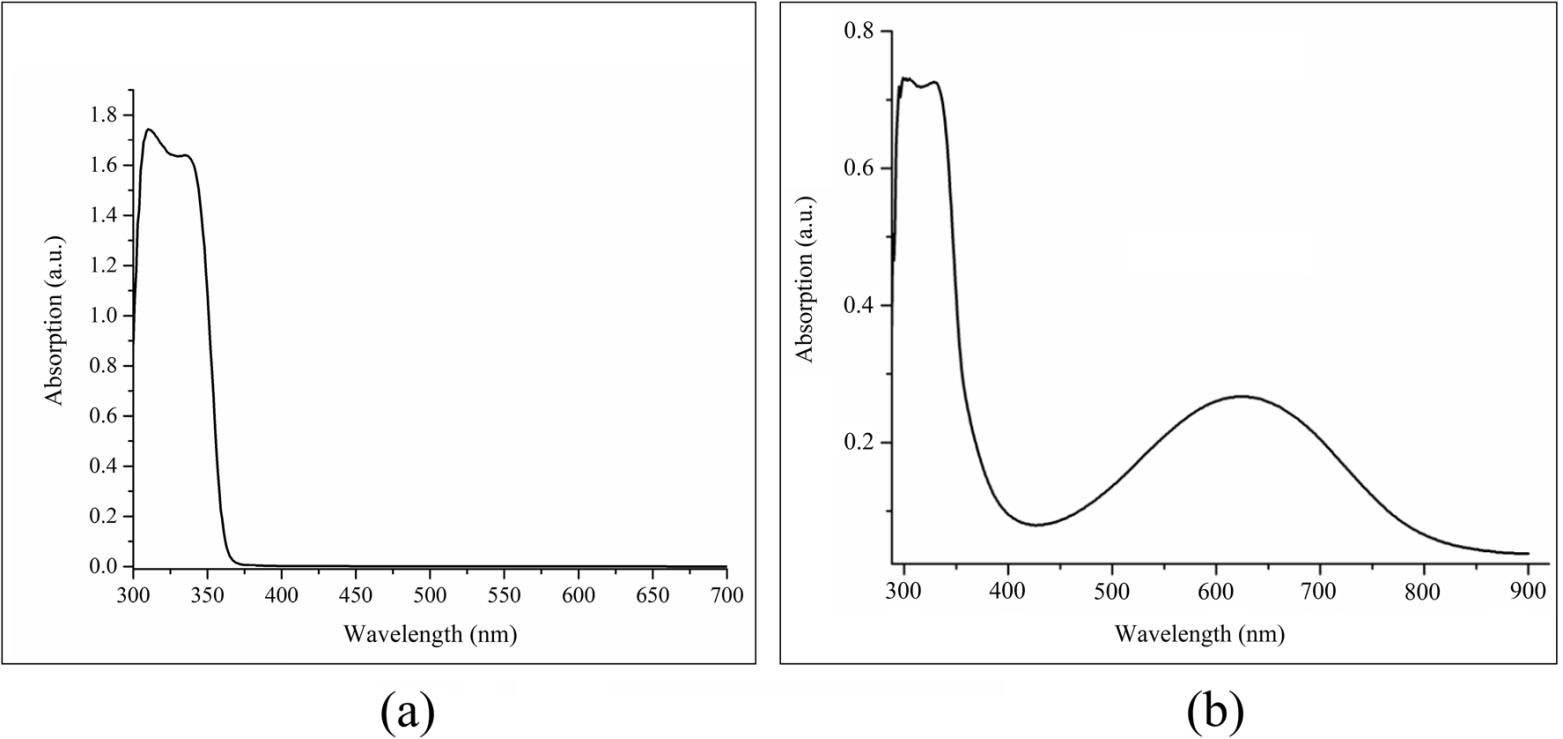
UV–Vis spectroscopy diagrams of (**a**) ZnO nanoparticles, and (**b**) PANI, at wavelength range of 250–850 nm.

In order to investigate the absorption and transmission properties of ZnO and PANI, their UV–Vis spectroscopy diagrams at wavelength range of 250–850 nm were obtained (Fig. 4). Figure 4a depicts the characteristic peaks of synthesized zinc oxide nanoparticles; one is an absorption peak at 350 nm which is related to free carriers’ absorption, and the other one is a peak at 310 nm which has resulted from exciton absorption of the sample^47^. Exciton absorption occurs at lower wavelengths. This is due to the nature of the ZnO nano-structure, which restricts the path of electron movement. Moreover, exciton absorption is higher than free carriers’ absorption as a result of Sommerfeld enhancement at the presence of Coulomb interaction. Figure 4b represents three characteristic peaks of PANI at 300, 340 and 630 nm; the first and the second are related to π-π* transitions, while the third is related to the polaron.

**Figure 5:**
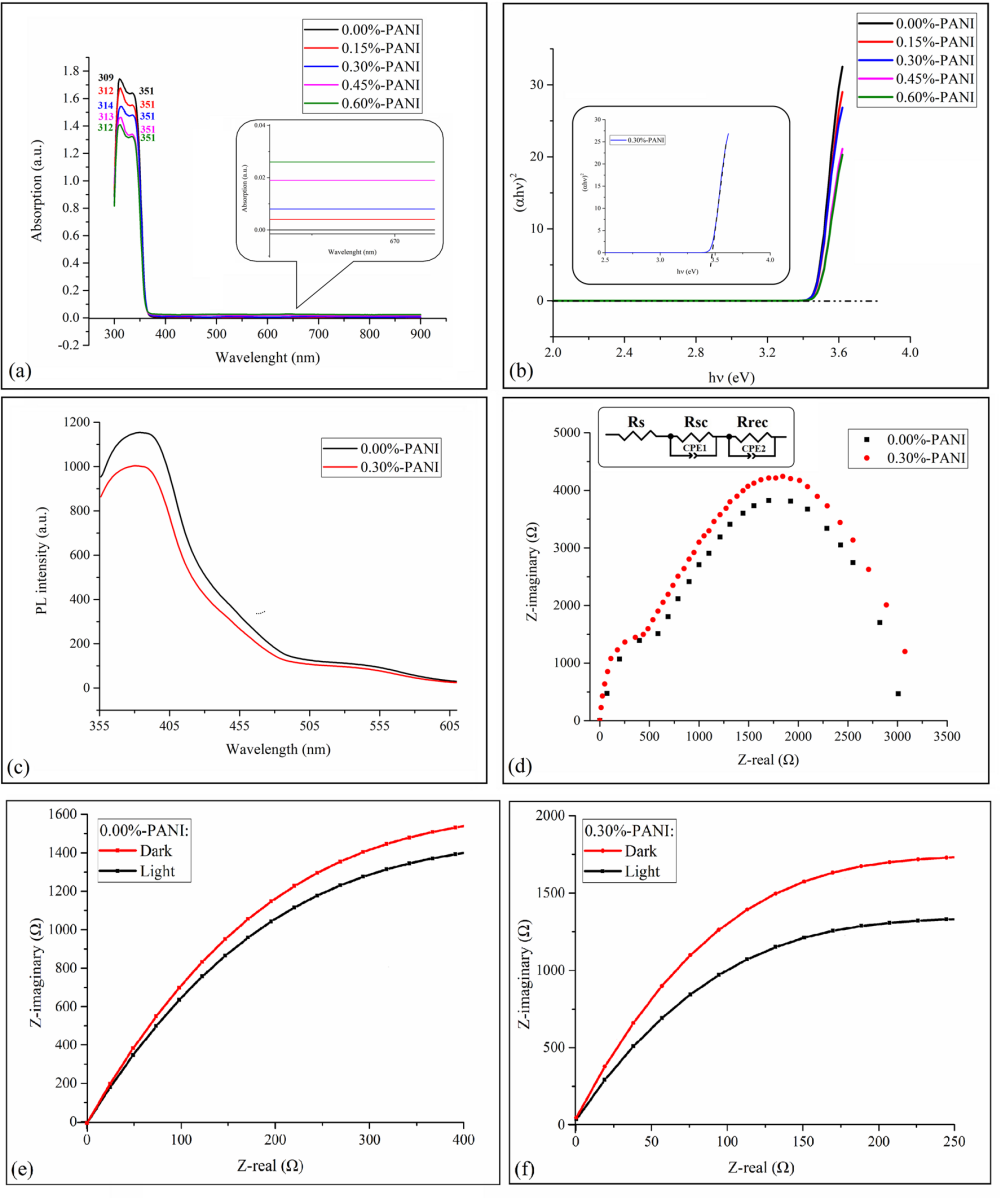
(**a**) UV–Vis absorption spectra of all samples, (**b**) Tauc diagram for estimating band gap of samples with different PANI percentages, (**c**) PL diagrams of pure ZnO nanoparticles and the 0.30%-PANI sample, (**d**) electrochemical impedance spectroscopy analyses of pure ZnO/NPs and the 0.30%-PANI sample, (**e**) dark–light electrochemical impedance spectroscopy analyses of 0.00%-PANI sample, and (**f**) dark–light electrochemical impedance spectroscopy analyses of 0.30%-PANI sample.

UV–Vis absorption spectra of samples with different PANI percentages, Fig. 5a, are indicative of the fact that at the wavelength region of 300–400 nm, where the peaks are mainly related to ZnO, the first peak shows a transition to longer wavelengths up to 0.30% of PANI, and then returns to shorter wavelengths. As discussed before, this peak is related to exciton absorption. In fact, the addition of PANI to ZnO facilitates exciton absorption, with the related wavelength shifting toward the visible region; accordingly, exciton production increases. However, as discussed before, further increase of PANI results in a higher probability of exciton’s electron and hole recombination which, in turn, means that higher energy is needed to obtain and maintain stable excitons. On the other hand, the second peak, which is related to ZnO bandgap absorption, is fixed at 351 nm. Furthermore, the optical band gap of the samples can be calculated by applying the Tauc model^48^ and the Davis and Mott model^49^ in the high absorbance region:

$$ \alpha {\text{h}}\nu = {\text{D}}\left( {{\text{h}}\nu {-}{\text{E}}_{{\text{g}}} } \right)^{{\text{n}}} $$

where hν is the incident photon energy, α is absorption, E_g_ is the optical band gap, and D is a constant. For a direct transition, n = 1/2 or 2/3, the former value is found to be more suitable for ZnO since it gives the best linear curve in the band-edge region^50,51^. Plotting the relationship between (αhν)^2^ and hν, the optical band gap of the samples is estimated; its value can be obtained by extrapolating the linear portion of the curve to the photon energy axis in the curve (Fig. 5b). As a good approximation, the obtained optical energy gap for all the samples is equal to 3.48 eV, which indicates the significant role of exciton absorption in the behavior of these photoelectrochemical cells. It is worth to say that according to the literature, the obtained optical band gap of pure ZnO-NPs is 3.52 eV^52^. In addition, at the wavelength region of 600–700 nm, the more the PANI, the more the sample’s absorption (the inset of Fig. 5a). Needless to say, at this wavelength region, PANI has a wide polaron peak. To explain further, it is interesting that although at wavelength region of less than 400 nm—where the absorption of ZnO is dominant—increasing PANI percentage results in less absorption. Indeed, the amount of absorption is an ascending function of PANI percentage at wavelength region of 600–700 nm.

It is well-known that an exciton has a finite lifetime, and electron–hole recombination results in photon emission. Hence, the photoluminescence (PL), defined as radiative recombination of excitons, is initiated by photo-excitation. Simply put, photoluminescence is the reversed form of absorption. According to PL diagram of pure ZnO (Fig. 5c), there are two peaks at wavelengths of ⁓ 375 nm and 520 nm; the first is related to the emission of energy gap-dependent excitation, and the second is attributed to the existence of single ionized oxygen vacancies. Besides, the PL diagram of the 0.30%-PANI sample, which has exhibited the best behavior in all previous steps, implies that compared with pure ZnO, PL intensity is lower for the composite. This means that the recombination rate in the composite is lower than in pure ZnO. In other words, the more the recombination, the higher the intensity of the PL spectrum.

In order to further investigate charge transformation and recombination processes, electrochemical impedance spectroscopy analysis was applied to both pure ZnO and 0.30%-PANI samples (Fig. 5d). Also, the equivalent circuit fitting was performed using the circuit presented in the inset of Fig. 5d. It should be noted that the EIS analysis has been exercised under illumination. Needless to say, R_s_ is the ohmic resistance of the system related to the substrate and electrodes. R_sc_, i.e., the “charge transfer resistance,” is the resistance of an electron against transference from/to the working electrode to the electrolyte; this parameter is presented by the first arc in Fig. 5d. Likewise, R_rec_, or the “recombination resistance,” is related to electron and hole transfer layers, and is presented by the second arc in Fig. 5d. R_s_ is the same for both samples since the ITO substrate and connecting wires are the same. Adding 0.30% of PANI causes R_sc_ to decrease from 585 to 438 Ω while R_rec_ increases from 2428 to 2650 Ω. This result signifies that charge transfer is getting easier and a faster electron transfer process occurs, whilst the recombination process is getting somehow difficult. Needless to say, charge transfer resistance decrement and recombination resistance increment result in better efficiency of the cells^53–55^. In other words, the analysis above does show that the 0.30% -PANI sample with lower charge transfer resistance has better bulk diffusion, which leads to higher stability. In contrast, the pure ZnO sample exhibits higher charge transfer resistance, indicating relatively lower bulk diffusion, which leads to lower stability^56^. Moreover, Figs. 5e,f show EIS measured in darkness for 0.00%-PANI and 0.30%-PANI samples, respectively. It is obvious that the radius of semicircles of both samples decreases after light illumination, indicating a lower R_sc_ resistance. In other words, charge transfer resistance of the samples under light illumination is lower than charge transfer resistance of the samples in the dark. This means that the photo-generated carriers can reduce the charge transfer impedance and, therefore, result in a faster electron transfer process at the electrode/electrolyte interface.

**Figure 6:**
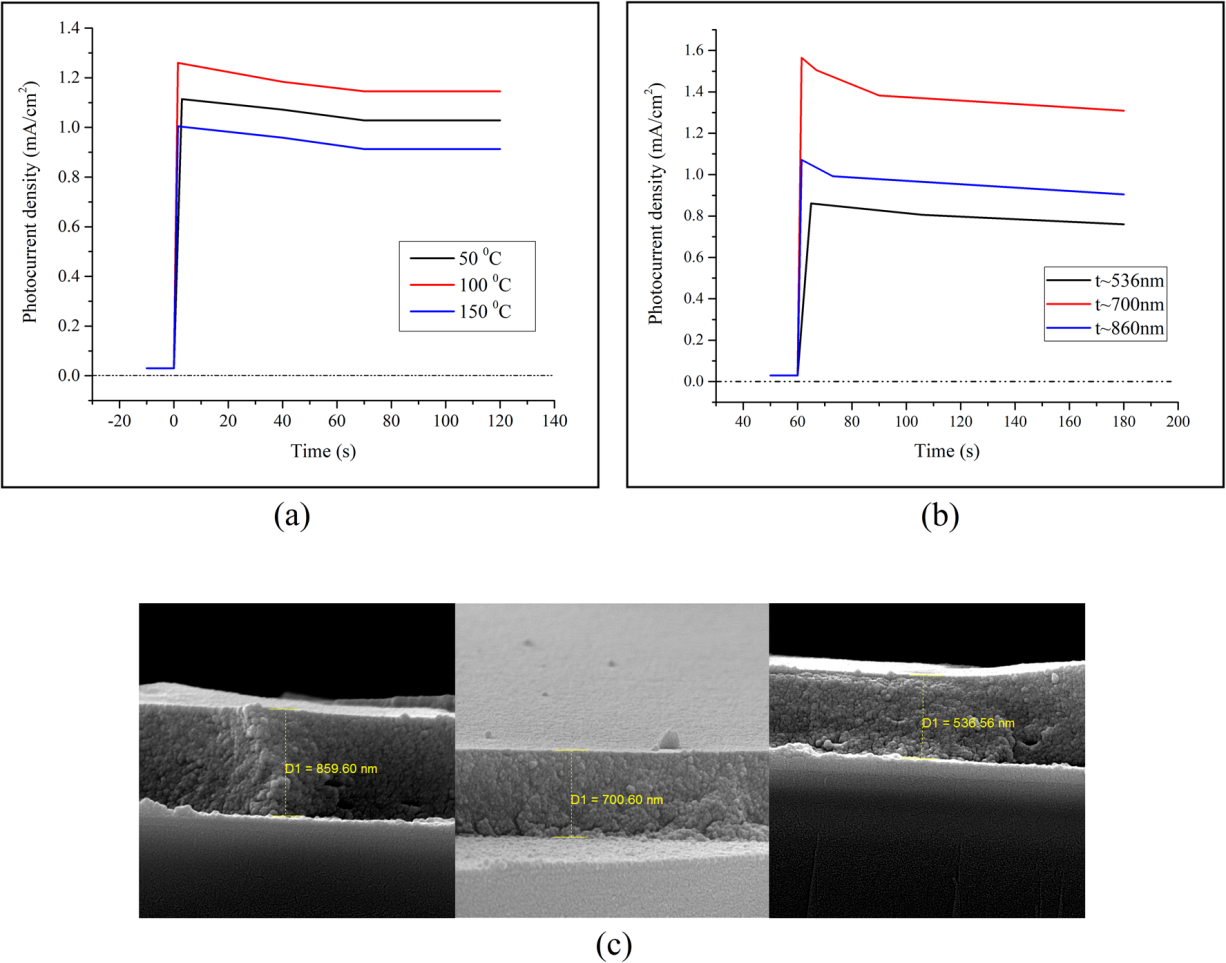
(**a**) The two electrode “photocurrent density- time” diagrams of different 0.30%-PANI samples, dried at temperatures of 50 °C, 100 °C and 150 °C for the same time interval, i.e., 30 min, (**b**) The two electrode “photocurrent density-time” diagrams of 0.30%-PANI samples with different thicknesses, dried at a temperatures of 100 °C, and (**c**) The indicative SEM micrographs of 0.30%-PANI samples with different thicknesses, dried at a temperatures of 100 °C.

Another investigation aimed at studying the effect of drying rate on the 0.30%-PANI sample. As above-mentioned, to reach this goal, three samples were dried at temperatures of 50 °C, 100 °C, and 150 °C for similar periods of time, i.e., 30 min. Regarding Fig. 6a, it is evident that the sample dried at 100 °C presents the highest photocurrent. An explanation for this behavior is that at low temperatures, the solvents have not been completely removed since the boiling point of NMP is ⁓ 203 °C^57^. Therefore, at a temperature of 50 °C, a solid layer has not yet formed, and thus, the related photocurrent is low. Moreover, photocurrent decrement at high temperatures could be attributed to oxidation of polyaniline because the process has been performed at ambient air conditions. According to the literature, the dependence of PEC cells’ behavior on drying and annealing temperature usually occurs in case of hybrid layers. One of the main reasons for such dependence is expressed as the improvement of the polymer’s chain order, which results in the improvement of the surface morphology of layer^58,59^. This, in turn, increases polymer crystallinity or relative orientation of polymer chains.

The effect of thickness was also studied on three different samples with 0.30% PANI, dried at a temperature of 100 °C. It is interesting that the layers’ thickness – whose photocurrent is the highest—displays an optimum at 700 nm, (Figs. 6b, c). Obviously, at lower thicknesses, where there are fewer charge carriers in the active layer, the sample’s resistance is high. At a thickness of 700 nm, there is an increase of photocurrent resulting from the charge carriers’ increment. Further increase in thickness produces extended path of electrons; as a result, the migration time interval of charge carriers exceeds their recombination time and, therefore, decreases photocurrent. In other words, thicker layers can contribute to the loss of charge collection, which, in turn, causes more recombination. Additionally, the dependence of the active layer’s behavior on its thickness may be affected by some other factors, such as mobility and lifetime of charge carriers as well as space charge^60^.

**Figure 7:**
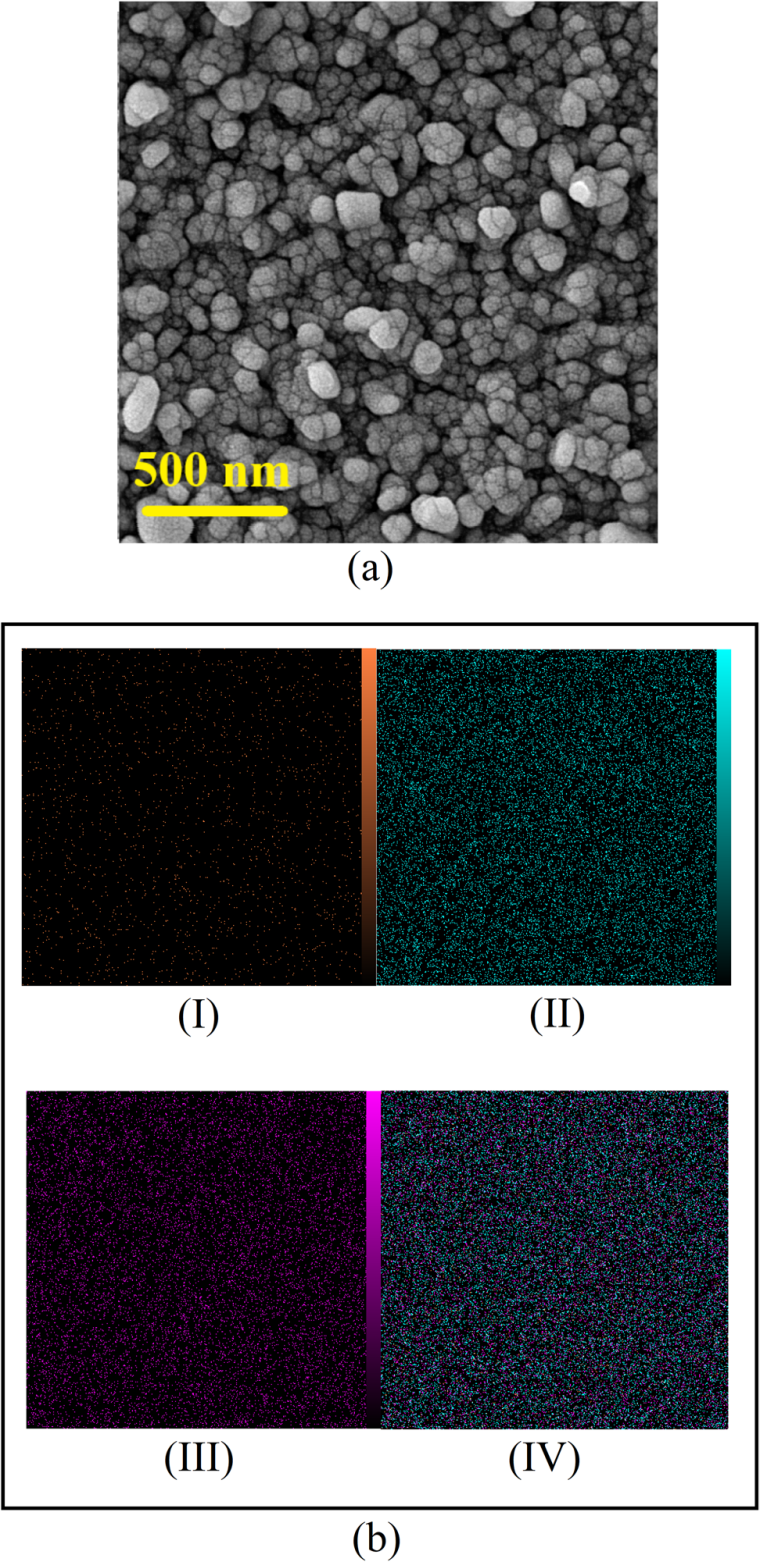
(**a**) SEM micrograph of the 0.30%-PANI sample, dried at a temperatures of 100 °C, with a thickness of 700 nm, and (**b**) EDS spot analysis images of 0.30%-PANI sample, which shows the distributions of zinc (I), oxygen (II), carbon (III), and EDS layered image (IV).

SEM micrographs could serve as the best and also the final confirmation of all the results previously obtained (Fig. 7a). Regarding SEM micrographs, it is evident that ZnO-NPs agglomeration forms ZnO grains, and PANI remains at the intergranular layer. Finally, EDS mapping of the well-behaved sample shows the distribution of ZnO as well as PANI particles (Fig. 7b). It goes without saying that PANI is homogeneously distributed between ZnO grains. Both SEM micrographs and EDS mapping are strong proofs of the previous discussions that electrons and holes transfer at inhomogeneous interfaces and that all of the previously stated results can be stabilized.

**Figure 8:**
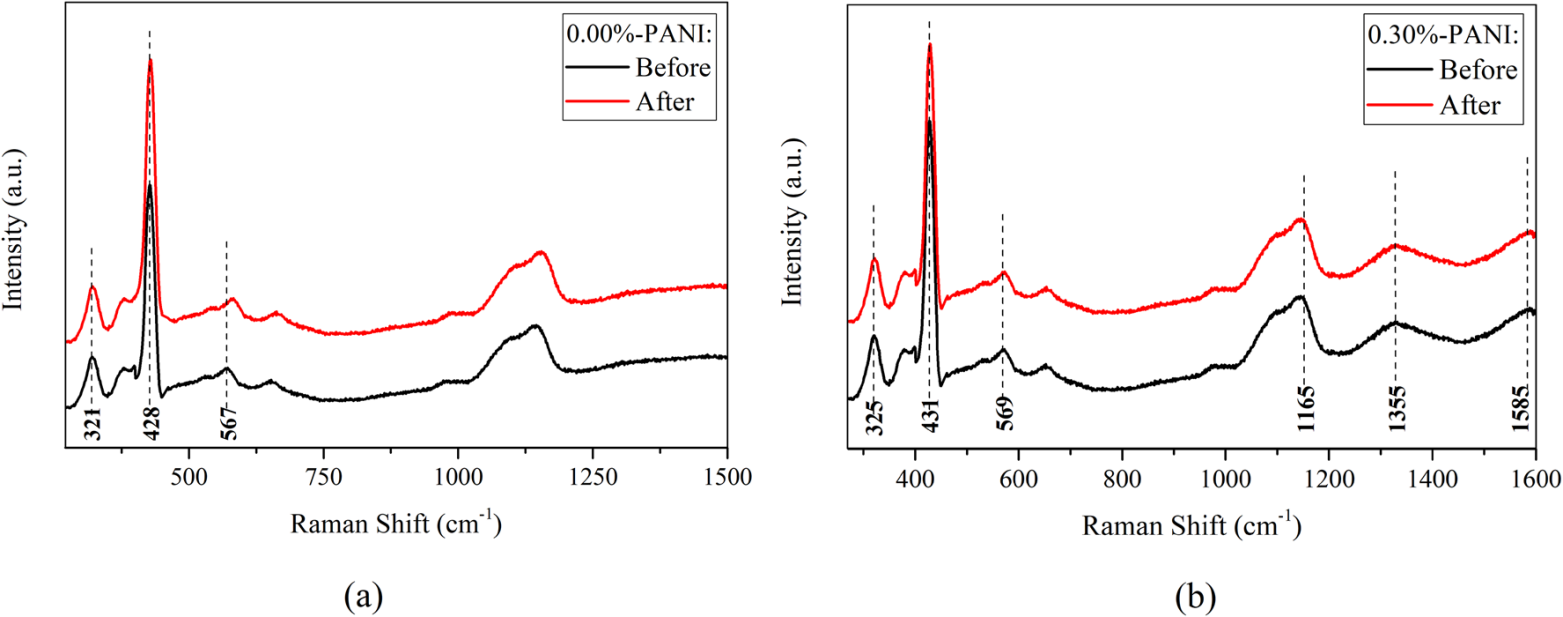
The Raman spectra of (**a**) 0.00%-PANI, and (**b**) 0.30%-PANI samples, before and after prolonged exposure.

Raman spectra of 0.00%-PANI and 0.30%-PANI samples, both before and after prolonged exposure, give insights into any structural changes. Figure 8a indicates that before irradiation, the Raman spectrum of 0.00%-PANI sample displays a high intensity peak appears at 428 cm^−1^ which is the characteristic peak of hexagonal ZnO for E_2_(high) non-polar vibration mode^32^. The Raman peak locates at 321 cm^−1^ assigns to the secondary Raman scattering arising from zero-boundary phonons of ZnO. In addition, a weak peak observed at ∼ 567 cm^−1^ of E_1_ (LO) mode can be related to oxygen deficiency in the sample. On the other hand, Raman spectrum of 0.30%-PANI sample before irradiation, Fig. 8b, demonstrates that composing ZnO-NPs with PANI causes the peaks of ZnO to endure a slight shift. The Raman peaks of PANI are also recognizable in this spectrum; the vibrations of C-H benzenoid or quinoid stretching, C=N quinoid stretching, and C=C quinoid stretching are represented by the peaks appeared at 1165 cm^−1^, 1355 cm^−1^, and, 1585 cm^−1^, respectively^32^. Then, the Raman spectra of both samples, after prolonged exposure, exhibit their modifications, causing Raman intensity to increase, and a slight wavenumber shift of particular peaks, as well. The partial change of the frequencies and relative intensities may reflect a decrease of the samples’ quality. Such behavior is obviously a consequence of changes in the bond structure.

## Conclusion

“ZnO-NPs/PANI” composite thin layers, spin-coated on ITO substrate, were used for water splitting via solar energy irradiation in order to produce renewable hydrogen from water. Findings indicate that the sample with 0.30% PANI displays the most beneficial performance since its photocurrent density is the highest. Besides, its photocurrent increment starts to increase at a voltage of ⁓ 1.23 V (vs. RHE), which is approximately in accordance with the theoretical potential of water electrolysis. Indeed, the “ZnO-NPs/PANI” inhomogeneous structure of this sample facilitates exciton generation via a shift in the related wavelength toward the visible region, and, hence, e–h production increases. As a result, it represents the highest photoelectrochemical efficiency due to the lowest rate of electron–hole recombination. This is a simple and low-cost method which introduces a compatible photocurrent with respect to previous studies. Besides, according to electrochemical impedance spectroscopy analysis, charge transfer of this sample is facilitated. Finally, the optimum drying temperature and layer thickness are obtained as 100 °C and 700 nm, respectively.

## Data Availability

The datasets used and analyzed during the current study are available upon request. If someone wants to request the data from this study, please contact “R.azmayesh@tabrizu.ac.ir”.
